# Uniphasic Blanching of the Fingers, Abnormal Capillaroscopy in Nonsymptomatic Digits, and Autoantibodies: Expanding Options to Increase the Level of Suspicion of Connective Tissue Diseases beyond the Classification of Raynaud's Phenomenon

**DOI:** 10.1155/2015/371960

**Published:** 2015-05-17

**Authors:** Francesca Ingegnoli, Roberta Gualtierotti, Annalisa Orenti, Tommaso Schioppo, Giovanni Marfia, Rolando Campanella, Claudio Mastaglio, Pier Luigi Meroni, Patrizia Boracchi

**Affiliations:** ^1^Department of Clinical Science & Community Health, University of Milano, 20122 Milan, Italy; ^2^Division of Rheumatology, Istituto Gaetano Pini, 20122 Milan, Italy; ^3^Medical Statistics and Biometry, 20122 Milan, Italy; ^4^Laboratory of Experimental Neurosurgery and Cell Therapy, Neurosurgery Unit, Fondazione IRCCS Ca' Granda Ospedale Maggiore Policlinico Milano, 20122 Milan, Italy; ^5^Rheumatology Unit, Ospedale Moriggia-Pelascini, 22015 Gravedona, Italy

## Abstract

In patients with Raynaud's phenomenon (RP), the role of medical history, capillaroscopy, and autoantibodies in order to provide an early diagnosis of connective tissue disease (CTD) were examined. 115 consecutive adults with uni-, bi-, or triphasic colour changes of the fingers were studied. RP was bilateral in 92.7% of patients. The middle finger was significantly more affected. A lack of association between fingers affected by RP and fingers with capillary abnormalities was observed OR = 0.75 (0.34–1.66). RP with the cyanotic phase had a higher risk at capillaroscopy to have hemorrhages OR = 4.46 (1.50–13.30) and giant capillaries OR = 24.85 (1.48–417.44). The thumb and triphasic involvement have an OR of 1.477 and 1.845, respectively. RP secondary to systemic sclerosis (SSc) had greater value of VAS pain (*p* = 0.011). The presence of anti-centromere antibodies was significantly associated with a higher risk of SSc (*p* < 0.001). 44.3% of subjects had uniphasic blanching of the fingers, and among these, 27% was diagnosed as having an overt or suspected CTD. Markers of a potential development of CTDs include severe RP symptoms, positive autoantibodies, and capillary abnormalities. These data support the proposal to not discharge patients with uniphasic blanching of the fingers to avoid missing the opportunity of an early diagnosis.

## 1. Introduction

Raynaud's phenomenon (RP) is a vasospastic response of the extremities to exposure to changes in temperature, emotional stress, or other reported triggers such as beta-blockers or smoking [1]. RP can be either primary (pRP) or secondary (sRP) to many nonrheumatic and rheumatic conditions. pRP is a benign idiopathic condition that should not progress, even if some studies suggested that between 12 and 20% of subjects with a diagnosis of pRP develop a sRP over time [2, 3]. By contrast, RP may be either a concomitant symptom that accompanies more specific clinical manifestations of nonrheumatic diseases or an early symptom of a developing connective tissue disease (CTD), such as systemic sclerosis (SSc), undifferentiated connective tissue disease (UCTD) or mixed connective tissue disease (MCTD), dermatomyositis (DM), systemic lupus erythematosus (SLE), Sjögren's syndrome (SS), or rheumatoid arthritis (RA).

RP frequently presents to physicians because of concerns about the possibility of an underlying disorder that can be associated with severe morbidity. The stratification of patients with RP is currently underpinned by the medical history, examination and investigation (i.e., capillaroscopy and antibodies) [1, 4, 5]. These findings may facilitate an effective screening and timely diagnosis.

It is generally accepted that diagnosis of RP is based on the history of at least two colour changes in the fingers [6, 7]. By contrast, patients with only the ischemic phase are excluded in these classifications as considered less severe. It has also been noted that the clinical characteristics of digital involvement are not uniform and may be useful to easily differentiate pRP from sRP. As an example, the thumb is less commonly affected than other digits and its involvement should be a warning for an underlying CTD [8, 9]. Moreover, other suspicious features are the severity of RP and the asymmetric involvement [10, 11].

Against this background, we investigated the role of medical history, capillaroscopy, and autoantibodies in differentiating between pRP and sRP in a cohort of patients with RP at the first rheumatologic evaluation. Our specific objectives were divided in two main steps:

- Before classifying patients in pRP or sRP, the main clinical characteristics of RP (i.e., symmetry, colour changes, number, and fingers affected by RP) and the associations of these characteristics with capillary abnormalities were examined.
- After classifying patients as pRP, sRP, or RP suspected secondary to CTD, the role of information easily obtained at the first medical evaluation that could be useful to differentiate these groups was investigated. Moreover, the role of autoantibodies in differentiating sRP versus RP suspected secondary to CTD was assessed.

## 2. Materials and Methods

### 2.1. Patient Selection and Assessments

Between February 2011 and May 2012, 115 consecutive adult subjects with RP at the first rheumatologic evaluation were recruited from two Italian rheumatology outpatient clinics (Division of Rheumatology, Gaetano Pini Hospital in Milano and Rheumatology Unit, Ospedale Moriggia-Pelascini in Gravedona). The study was approved by both ethics committees and informed consent was obtained from all patients. During a comprehensive baseline evaluation data were collected from medical history, diagnostic examination, and investigation as described below.

RP was defined as repeated, reversible vasospastic episodes of ischemia of the digits upon exposure to cold and/or in association with emotional stress and characterised by blanching, possibly followed by cyanosis and/or postischemic red flushing upon rewarming. Patients with uni-, bi-, or triphasic colour changes were included in the study. Because the screening programme for RP is made by basic and affordable procedures, we decided to include in the definition of RP even patients with uniphasic blanching of the fingers. We have presumably little to lose and much to gain from early detection of CTD.

The episodes could have been accompanied by varying degrees of paresthesia, numbness, or pain. Patients were also asked to complete a self-reported questionnaire which enquired about the fingers affected by RP, the colour changes, and visual analogue scales (VAS) to assess pain, discomfort, and severity of RP. Full medical history (e.g., current drug treatment, exposure to toxic agents, occupational history, and family history) and symptoms pointing to an underlying CTD (e.g., pitting scars, gangrene, sclerodactyly, photosensitivity, rashes, telangiectasias, orogenital ulcers, xerophthalmia, episcleritis, scleritis, uveitis, arthralgias, arthritis, myalgia, muscle weakness, fatigue, serositis, dyspnea, pulmonary fibrosis and/or hypertension, heart ischemia, conduction disturbance, nephrotic syndrome, glomerulonephritis, leukopenia, anemia, thrombocytopenia, xerostomia, dysphagia, esophageal hypomotility, or neuropathy) were taken. All subjects underwent complete examination with particular focus on digital ulceration, pitting scars, and sclerodactyly.

In both centers, nailfold videocapillaroscopy was performed using equipment with a 200x optical probe, with the images being captured, coded, and stored using Videocap software (DS-Medica, Milano, Italy). All of the recordings were made with the subject in a sitting position, with temperature of 22 to 25°C and with their hands at heart level. The procedure was explained and a drop of immersion oil was applied to the nailfold to maximize the translucency of the keratin layer. In each of the subjects all the fingers of both hands were examined. In each center, the capillaroscopy images were evaluated by an experienced observer (C. M. at the Ospedale Moriggia-Pelascini and R. G. at the University of Milan). Then, all the images were reviewed by a third observer (F. I. at the University of Milan). The agreement among observers was tested in a previous study [12]. For each image, the presence of giant capillaries, neoangiogenesis, microhemorrhages, and avascular areas were evaluated as described previously [12]. The overall capillaroscopy pattern was classified as within the normal range [13] or as abnormal capillaroscopy pattern when the following capillary abnormalities were present: microhemorrhages, giant capillaries, neoangiogenesis, and decrease in the capillary number [12].

Serum and plasma samples were collected and stored at −80°C until assayed in the same laboratory for anti-nuclear antibodies (ANA) by indirect immunofluorescence on HEp2 cells (Antibodies Inc., Davis CA), considering positive those samples with a dilution ≥1 : 160 [14]; anti-dsDNA; antiextractable nuclear antigens (anti-ENA). The autoantigens tested for anti-ENA were the following: U1RNP, Sm, Ro52, La, Jo-1, Rib-P, PCNA, Scl-70, CENP, Fibrillarin, RNA Pol III, PM-Scl, and Mi-2 by EliA (ImmunoCap250, Phadia, Freiburg, Germany); Scl-70, CENP A, CENP B, RP11, RP155, FIBRILLARIN, NOR90, TH/T0, PM-Scl100, PM-Scl75, ku, and PDGFR, Ro52 by (DotBlot EUROLINE Systemic Sclerosis EUROIMMUN AG Luebeck, Germany); and Mi2, ku, PM-Scl100, PM-Scl75, Jo1, SRP, EJ, OJ, PL7, PL12, and Ro52 by DotBlot Myositis profile IgG (EUROIMMUN AG Luebeck, Germany).

Based on the above-mentioned set of diagnostic examination and investigation, patients with RP were classified as (1) pRP (i.e., normal capillaroscopy, negative ANA, and anti-ENA, in the absence of any symptoms/signs suggestive of CTDs); (2) RP secondary to suspected CTD (i.e., abnormal capillaroscopy or positive autoantibodies—ANA and/or anti-ENA—, and without any symptoms/signs suggestive for CTDs); (3) sRP when RP was associated with an overt CTD that fulfilled the related criteria of SSc [15], or MCTD [16], or SLE [17], or SS [18], or RA [19].

### 2.2. Data Analysis

A mixed logistic regression model, considering each finger as an experimental unit, was used to evaluate the association among the presence/absence of RP and the finger/hand affected by RP (model I), the association among presence/absence of capillary abnormalities, and the presence/absence of RP on finger/hand affected by RP and RP colour changes (model II). For each model, the response was a 0/1 dummy variable (coded 1 if the finger was affected by RP in model I and coded 1 if the finger was affected by capillary abnormalities in model II). In both models, fingers and hands were included as dummy variables (reference: middle finger and left hand, resp.). In model II, RP severity and each of the three colour phases are included as dummy variables. To account for the correlation among measures within the same subject, a variable identifying each patient was included in the model as random effect, whereas all the mentioned variables were included as fixed effects.

Then, patients were classified, as described in the above paragraph, in pRP, RP secondary to suspected CTD and sRP to overt CTD.

Logistic regression models, considering the patient as experimental unit, were applied for the analysis of the association among different diagnosis (i.e., pRP versus RP secondary to suspected CTD and RP secondary to suspected CTD versus sRP to overt CTD) and clinical features (i.e., RP duration, thumb affected by RP, number of fingers affected by RP, number of RP colour phases, VAS pain, VAS severity, and VAS discomfort). All the variables included as mandatory in the classification criteria were excluded from the analysis [7, 15, 20]. For example, in patients with pRP, according to classification criteria [7, 20], nailfold capillaroscopy must be always within the normal range, and autoantibodies must always be negative. Therefore, these two variables were considered only in the analysis of RP secondary to suspected CTD versus sRP to overt CTD. Moreover, concerning autoantibodies, only ANA were considered in the statistical analysis as in clinical practice ANAs are considered a first-line investigation.

Each model (i.e., pRP versus RP secondary to suspected CTD and RP secondary to suspected CTD versus sRP to overt CTD) was analyzed separately, as a different set of variables were considered in each model.

As first step, univariate analysis was performed. Then, in the multivariable model building phase, the ratio between the number of subjects in each categories and the number of regressors was taken into account [21]. A maximum of six regressors was considered for the model concerning pRP versus RP secondary to suspected CTD, and a maximum of four regressors was considered for the model RP secondary to suspected CTD versus SSc. The choice of the model was based on the best subset procedure [22].

Statistical analysis was carried out using software R, with packages reshape, lme4 and bestglm added.

## 3. Results

### 3.1. Clinical and Capillaroscopy Features of Patients with RP at the First Evaluation

One hundred and fifteen consecutive subjects with RP (median age 45.2 yrs, 105 women) at their first medical evaluation were enrolled in this study. All the patients were Caucasians. RP was bilateral in 102 (92.7%) of patients.

RP was reported with similar frequency on the fingers of the right and left hand, respectively: middle fingers (95.5% and 88.2%), forefingers (85.5% and 84.5%), ring fingers (86.4% and 83.6%), little fingers (68.2% and 66.4%), and thumbs (39.1% on both hands). The middle finger was significantly more affected than all the other fingers. Considering middle finger as reference, the following OR (95% CI) were estimated: forefinger OR = 0.33 (0.15–0.72), ring finger OR = 0.33 (0.15–0.72), and little finger OR = 0.05 (0.02–0.11).

Moreover, classic triphasic colour changes were present in only 10.5% of subjects, two colour changes in 45.2% and one colour change in 44.3% of cases. The blue phase due to deoxygenation of venous blood was less frequently observed compared to the white or red phases.

In patients with capillary abnormalities, the frequency of distribution of nailfold capillary abnormalities was similar on the fingers of the right and left hand: middle fingers (24.3% and 25.2%), forefingers (14.8% and 17.5%), ring fingers (29.6% and 26.1%), little fingers (20.9% and 15.7%), and thumbs (14.3% on both hands). Capillary abnormalities were significantly more frequent on the middle finger than on the forefinger and little finger.

Considering single fingers, capillary abnormalities were observed in the 20% of fingers not affected by RP and in the 23% of fingers affected by RP. A mixed effects model analysis confirmed the lack of association between fingers affected by RP and fingers with capillary abnormalities; in other words, fingers affected by RP were not significantly associated with a greater risk to have capillary abnormalities OR = 0.75 (0.34–1.66).

Moreover, it has been observed that capillary abnormalities (i.e., microhemorrhages and giant capillaries) were more frequently found in the fingers with RP cyanotic phase OR = 8.66 (2.32–32.27). Considering middle finger as reference, the following OR (95% CI) were estimated: forefinger OR = 0.37 (0.20–0.70), ring finger OR = 1.32 (0.74–2.35), and little finger OR = 0.42 (0.22–0.81).

Among different capillary abnormalities, microhemorrhages were the most commonly reported. By singly modelling different capillary abnormalities it was noted that fingers affected by RP with the cyanotic phase were associated with a higher risk of microhemorrhages OR = 4.46 (1.50–13.30) and giant capillaries OR = 24.85 (1.48–417.44).

### 3.2. Role of History, Examination, and Investigations in the Differential Diagnosis of RP

Based on the above-mentioned set of diagnostic examination and investigation (i.e., clinical symptoms, capillaroscopy, and autoantibodies), 39 patients were classified as pRP (median age 44.8 yrs, 38 women), 20 SSc (median age 58.2 yrs, 15 women), 1 woman having MCTD, and 55 RP secondary to suspected CTDs (median age 42.4 yrs, 51 women). 17 patients with SSc (85%) were positive for both ANA and anti-ENA, while 29 patients with RP secondary to suspected CTD (52.7%) were positive for ANA and 22 for anti-ENA (40%). CENP A/B and Ro52 were the most common specificities observed in patients with SSc (47.05% and 17.6%, resp.) and in patients with RP secondary to suspected CTD (31.8% and 36.7%). Demographic data, clinical features, and diagnostic investigations are summarised in Table 1.

To evaluate the usefulness of each variable in the differential diagnosis between pRP versus RP secondary to a suspected CTD the binomial logistic regression analysis was applied (Table 2). As mentioned above, all the variables included as mandatory in the classification criteria of pRP (i.e., ANA and capillaroscopy that must be negative) were excluded from the analysis. None of the variables examined was significantly associated with a greater or lesser risk to be classified as pRP or RP secondary to a suspected CTD (Table 2). These results were confirmed in multivariable analysis, since no models were identified by the best subset analysis.

The results of the univariate analysis for the comparison of RP secondary to suspected CTDs versus SSc are reported in Table 3(A). Only ANA positivity with anticentromere pattern was a significant factor to discriminate between the two groups (*p* value < 0.001). Concerning multivariable analysis, results of the best subset model are reported in Table 3(B). Patients with SSc had significantly greater value of VAS pain (*p* value = 0.011). The presence of ANA with anticentromere pattern was significantly associated with a higher risk of being classified as SSc (*p* value < 0.001).

## 4. Discussion

The screening approach used in this study based on medical history, nailfold capillaroscopy, and autoantibodies may provide a window of opportunity for therapeutic intervention, a potential chance to slow or block disease progression, and permit early monitoring of these patients.

Community-based studies showed that RP is a common problem in women, with a prevalence ranging from 4 to 21% in the population [2, 23–26], supporting the need of an easy and affordable algorithm to differentiate primary from secondary forms.

In agreement with previous studies [2, 23, 25, 27–30], in our cohort RP was more prevalent among women than men. By contrast, the median age of patients classified as pRP (44.2 years) was older than other studies, in which the “late-onset” RP (over 40 years old) was reported in a percentage of cases ranging from 22 to 40% [28, 31, 32]. This could be due to the fact that the patients' recruitment was done in tertiary referral hospitals. Only a long-term follow-up will clarify if these patients are really pRP or as reported they are at risk of developing CTDs [31].

For a more comprehensive evaluation of RP in this study, we included patients even with uniphasic blanching of the fingers if well documented, while the other questionnaires select only patients with two or three colour changes of the fingers [6, 7]. We agree with the statement [20] that “uniphasic” RP does not carry the medical significance of biphasic or triphasic symptoms, but based on our results, 44.3% of subjects had uniphasic blanching of the fingers, and among these, 27% was diagnosed as having an overt CTD or a suspected CTD based on physical examination, capillaroscopy, and autoantibody profile. These data support the proposal to not discharge patients with uniphasic blanching of the fingers from further evaluations.

We observed that RP was bilateral in the majority of patients, and the middle finger was more frequently involved. A decreasing frequency of RP from the middle finger (95.5%–88.2%) to the thumb (39.1%) was noted, confirming that the thumb involvement is rare [8, 9]. Previous studies proposed thumb involvement as a red flag for secondary RP [8, 9]. Although our results showed that thumb involvement and three colour changes of RP were not able to discriminate between pRP and RP secondary to a suspected CTD, it should be noted that the OR was 1.47 and 1.85, respectively. These data may be due to the small group of patients examined and may suggest that further studies with a larger series of patients are needed.

When present, nailfold capillary abnormalities were equally observed in both hands and in all the fingers, even if more frequently in the middle finger. They were present in fingers affected and not affected by RP. These results support the recommendation to perform capillaroscopy on all the fingers, because microvascular changes are not related to the presence of RP in the finger examined. Moreover, microhemorrhages are the more frequent abnormalities among microvascular changes. The presence of giant capillaries and microhemorrhages were more frequently observed in patients with the blue phase of RP.

The role of nailfold capillaroscopy is strongly supported by our data. Even if we use a videocapillaroscope, this investigation can be performed with different equipment, such as dermatoscope that has been demonstrated to be comparable to the videocapillaroscope [33].

Concerning autoantibodies, in our cohort, only ANAs with anticentromere specificity were associated with a significant high risk of developing SSc, thus confirming previous data from the medical literature [34, 35]. Moreover, previous studies suggest the role of anti-PM75 for identifying patients with more severe clinical features and anti-PM100 for patients with an overlap syndrome [36, 37]. In this study, we found anti-PM75 only in patients with RP secondary to suspected CTD and anti-PM100 only in patients with overt SSc, but only a strict follow-up of these patients could confirm this hypothesis. In agreement with previous studies [38, 39], anti-Ro52 was the most common autoantibody detected, but none of our patients had other clinical manifestations except for RP or skin involvement for the group with overt SSc.

RP may predate the development of CTD by many years, and markers for identification of patients with RP secondary to a suspected CTD include severe RP symptom (high VAS pain), autoantibody positivity, and capillary abnormalities. The presence of three colour changes and the thumb involvement might be a warning for secondary forms. These data may help physician to identify high-risk patients for whom closer and further medical examination may be necessary.

## 5. Conclusions

It is generally accepted that the decision to screen patients with RP should never be made lightly. In this field, nailfold capillaroscopy is a key investigation. We reported that microvascular abnormalities assessed are not necessarily present on the fingers affected by RP, thus supporting the recommendation to perform capillaroscopy on all the fingers that before was supported only by the experience of the examiner. Positivity for autoantibodies (e.g., anti-centromere antibodies) may also help in discriminating pRP from sRP.

Faced with a multitude of laboratory and diagnostic options, this study adds some useful tips to improve the screening of RP with a combined affordable approach. We suggest that the medical history should be focused also on clinical features of RP. In particular, secondary forms are more painful and characterized more often by the presence of three colour changes and the involvement of the thumb.

For the first time, we showed that the inclusion in the screening and follow-up programmes of all patients with a vasospastic response of the extremities, even with uniphasic blanching of the fingers, can improve the very early detection of CTDs.

## Figures and Tables

**Table 1:** Demographic data, clinical features, and diagnostic investigations of the study cohort.

	Primary RP	RP secondary to suspected CTD	Systemic sclerosis^*^
Number of subjects	39	55	20
Female number (%)	38 (97.4%)	51 (92.7%)	15 (75%)
Age median (IQR) years	44.8 (31.7–53.8)	42.4 (31.3–52.9)	58.2 (47.2–68.8)
RP duration median (IQR) years	5 (1.8–10)	3 (1.1–10)	2 (1.3–3)
RP bilateral number (%)	36 (92.3%)	47 (94%)	18 (90%)
Number of fingers with RP median (IQR)	8 (6–10)	8 (6–10)	8 (7.5–10)
Thumb affected by RP	15 (38.5%)	22 (44%)	7 (35%)
Uniphasic RP number (%)	17 (43.7%)	20 (36.3%)	11 (55%)
(i) White number (%)	15 (38.5%)	19 (34.5%)	10 (50%)
(ii) Blue number (%)	1 (2.6%)	1 (1.8%)	1 (5%)
(iii) Red number (%)	1 (2.6%)	0 (0%)	0 (0%)
Biphasic RP number (%)	18 (46.1%)	29 (52.8%)	5 (25%)
(i) White/blue number (%)	2 (5.1%)	4 (7.3%)	3 (15%)
(ii) White/red number (%)	16 (41%)	25 (45.5%)	2 (10%)
(iii) Blue/red number (%)	0 (0%)	0 (0%)	0 (0%)
Triphasic RP number (%)	2 (5.1%)	6 (10.9%)	4 (20%)
VAS pain median (IQR)	12 (1–45)	13 (0–41.25)	40 (6.1–47.4)
VAS severity median (IQR)	13 (4–47.5)	15 (0–36.5)	9 (0–37)
VAS discomfort median (IQR)	2 (0.5–6.5)	3 (0–5)	3 (1–5.5)
ANA positivity number (%)	0 (0%)	29 (52.7%)	17 (85%)
Anti-ENA positivity number (%)	0 (0%)	22 (40%)	17 (85%)
Antigen		7 CENP A and B	8 CENP A and B
		8 Ro52	2 Scl70
		2 Scl-70; 1 Th/To	3 Ro52; 3 PM100
		2 PM75; 2 Ku;	1 Th/To; 1 Ku;
		3 PL7; 1 OJ; 1 EJ	1 RP155; 1 PL7
Capillaroscopy abnormal number (%)	0 (0%)	6 (10.9%)	16 (80%)

^*^In our cohort, all the patients with sRP were diagnosed as systemic sclerosis.

RP: Raynaud phenomenon; CTD: connective tissue disease; IQR: interquartile range; ANA: anti-nuclear antibodies; anti-ENA: antiextractable nuclear antigen; VAS: visual analogue scale.

**Table 2:** Univariate analysis of primary RP versus RP secondary to suspected connective tissue disease. All the variables included as mandatory in the classification criteria of primary RP (i.e., ANA and capillaroscopy that must be negative) were excluded from the analysis.

Primary RP versus RP secondary to suspected connective tissue disease		
	OR (95% CI)	*p* value
RP duration (years)	0.999 (0.995–1.003)	0.688
Number of fingers affected by RP	1.031 (0.846–1.255)	0.764
Thumb affected by RP	1.477 (0.535–4.078)	0.452
Number of RP colour phases	1.845 (0.864–3.94)	0.114
VAS pain	0.996 (0.975–1.018)	0.742
VAS severity	0.997 (0.978–1.016)	0.756
VAS discomfort	0.964 (0.821–1.133)	0.659

RP: Raynaud phenomenon; OR: odds ratio; CI: confidence interval; VAS: visual analogue scale.

**Table 3:** Univariate analysis of Raynaud's phenomenon secondary to a suspected connective tissue disease versus systemic sclerosis (A) and association of different variables assessed by binomial logistic regression (B).

RP suspected secondary to a connective tissue disease versus systemic sclerosis		
	OR (95% CI)	*p* value
(A) Univariate analysis		
RP duration (years)	0.993 (0.984–1.002)	0.133
Number of fingers affected by RP	1.04 (0.843–1.284)	0.713
Thumb affected by RP	0.587 (0.192–1.795)	0.351
Number of RP colour phases	0.634 (0.279–1.438)	0.275
VAS pain	1.011 (0.99–1.033)	0.305
VAS severity	0.996 (0.975–1.018)	0.738
VAS discomfort	1.079 (0.889–1.309)	0.443
ANA positive (nonanticentromere) versus ANA negative	2.652 (0.61–11.518)	0.193
ANA positive (anticentromere) versus ANA negative	25 (4.248–147.122)	<0.001

(B) Binomial logistic regression		
VAS pain	1.067 (1.015–1.122)	0.011
VAS severity	0.955 (0.912–0.999)	0.049
ANA positive (nonanticentromere) versus ANA negative	2.456 (0.499–12.088)	0.269
ANA positive (anticentromere) versus ANA negative	49.023 (6.566–366.005)	<0.001

RP: Raynaud phenomenon; ANA: anti-nuclear antibodies; VAS: visual analogue scale; OR: odds ratio; CI: confidence interval.
